# The Student as the Nurse Sees Him

**Published:** 1889-09-14

**Authors:** 


### The Student as the Nurse Sees Him.

I was rather startled at being asked my opinion of students. I really had never formulated any opinion about them. I have had students about me during the whole of my nursing career, and it is really going too far back into the mists of antiquity to recall how I felt towards them in my probationer days. Students are, to me, such an integral part of hospital life, that to be asked my opinion of them as a whole is like being asked my opinion of daylight, or any other essential familiar fact of life.

Still, since I am asked the question, I must strive to answer it. With the outer life of the student I have nothing to do; I don't profess to know him at home or in the class-room, or anywhere but in the wards. There I must speak of him with almost unqualified approbation. Of course there are individual differences. One can't help perceiving—we nurses are taught to use our eyes more than our tongues—that Mr. Brown has been used to different society from that Mr. Robinson is familiar with, and that Mr. Smith is quicker at his work than Mr. Jones. But one sees at the same time that Mr. Robinson feels as true a respect for the nurses and the patients as anybody else, and that Mr. Jones is thoroughly anxious to do his work well. I spoke of respect for the patients. Yes, I consider that essential. I can't imagine any man who has not a real feeling of tenderness for humanity, especially suffering humanity, simply as such, making a good doctor; and of such we see little in the wards. No doubt such men exist among medical students, and come round with their teachers, but they do not seek those positions as dressers and clerks that are so eagerly coveted by most students. But I have never known any forget for a moment the reverence due to the suffering before them, or to the presence of women.

On the latter point I will simply say that I have always found the students I have come in contact with courteous, respectful, and ready to help the nurses in every way in their power. It is said that a nurse does not get such good training at a hospital where they are many students; the latter claim the right to do all dressings and the like, leaving the nurse no scientific work to do. Where students are very numerous this may occasionally be the case, and it might be necessary for a nurse who is going into private surgical work to supplement her ordinary training by a few months in a hospital with no medical school attached; but on the whole I do not think there is much foundation for the complaint. In very few hospitals is the number of students so disproportionately large, and in none does either student or house surgeon trench upon the work of nursing, strictly so-called. Of course, where the nurse wants to set fractures there is sure to be trouble just as there would be if the house surgeon claimed the right to make beds, but such invasion of each other's dominions is too rare on both sides to be worth discussing. Remembering the true relations of doctor and nurse, I feel convinced that nothing but good can result from their learning their professions side by side. There are many dressings which it is in every way better should be done by men, and so far from there being a struggle for the work, I occasionally find it necessary to indicate to a nurse when she should go forward and offer her aid. Any disputes that arise between sister and house surgeon, nurse and student, are due to the idiosyncrasies of individual characters, and not to anything essential in the nature of their work.

With regard to the relations of students and patients, I think the intercourse is good for both. The staff physicians and surgeons are busy men; they do their work well, never grudging necessary time; but they cannot give more than the attention necessary to the case. Now the student looks after the case, and the patient, too. It is wonderful what tact those young men—little more than boys, many of them—develop in dealing with the queer, cantankerous bits of humanity we get in hospital. That must be good for the students—good for them as practitioners and good for them as men. All patients want a little managing, whether they are old or young, and the art of eliciting symptoms and constitutional histories under cover of a gossip is often learned, almost unconsciously, in the wards. Then the morbid shyness or unreasoning terror which patients often display on their first entrance has to be overcome; so many of those who come to us, both children and adults, having had so much more cause to fear than to love and trust their fellow-mortals. On the other hand, the students cheer up the patients. Their visits are a change in the inevitable monotony of hospital life. First there is the interest attaching to the dressing or the auscultation, painful perhaps, but of the nature of a mild excitement—a thing which has its own value. We are all alike in feeling better if a little fuss is made about us. You remember *Punch's* street arab who says, "Thank you, sir, that done me a lot of good," when the doctor takes his temperature. And no doubt the poor fellow did feel better for the moment. Then the student brings with him a bit of the outer world; his cheery face is like a breath of fresh air, and as a rule he has a pleasant word for the patients under his charge. It is good for people, even if they have lost heart and hope themselves, to meet with those who are young and hopeful; youth is like sunshine, one can't feel utterly depressed under its influence.

You would be surprised to see the really friendly feeling that often springs up between students and patients. When the patient is convalescent and can creep out into our garden, you will find the students out talking to them, perhaps taking a man some tobacco, or a woman an illustrated paper; in some way helping to make things brighter for them. As for the child-patients, of course, they like their young doctors, though, perhaps, they get a little jealous of special attentions paid to each other. I certainly think the little rogues profit a good deal by the presence of students. I can give you an instance of that. One of our convalescent children left us yesterday; where do you think she was going? One of our students took her to spend a week or two in the country with his mother and sister—took her himself, I mean. How would you like a railway journey with an invalid child of three years old?

In conclusion, I must say that I think medical students get a much worse reputation than they deserve. We have our classic pictures of them, and our standard jokes at their expense. Perhaps forty years ago they were mostly rough and rowdy. I don't know; but I am sure that the vast majority of them are not so now. Among the students at this hospital are one or two men who have been at a university before they took up medicine, and their culture affects the others. Those whose home influences are refined naturally show traces of these in hospital life, and those who haven't much to help them in private life gradually lose many asperities by contact with men of higher type, so I will venture to say that medical students, as a whole, are a hard-working, good-natured, and kind-hearted class, who, so far, at least, as we nurses can judge, have in them the making of good and conscientious practitioners.

